# Profiling of phytohormones in apple fruit and buds regarding their role as potential regulators of flower bud formation

**DOI:** 10.1093/treephys/tpac083

**Published:** 2022-08-11

**Authors:** Anton Milyaev, Julian Kofler, Yudelsy Antonia Tandron Moya, Janne Lempe, Dario Stefanelli, Magda-Viola Hanke, Henryk Flachowsky, Nicolaus von Wirén, Jens-Norbert Wünsche

**Affiliations:** Institute of Crop Science, Section of Crop Physiology of Specialty Crops (340f), University of Hohenheim, Emil-Wolff-Street 25, 70599 Stuttgart, Germany; Institute of Crop Science, Section of Crop Physiology of Specialty Crops (340f), University of Hohenheim, Emil-Wolff-Street 25, 70599 Stuttgart, Germany; Department Molecular Plant Nutrition, Leibniz Institute of Plant Genetics and Crop Plant Research (IPK), Corrensstr. 3, 06466 Stadt Seeland, OT Gatersleben, Germany; Julius Kühn-Institute (JKI), Institute for Breeding Research on Fruit Crops, Federal Research Centre for Cultivated Plants, Pillnitzer Platz 3a, 01326 Dresden, Germany; Department of Primary Industries and Regional Development, Government of Western Australia, Locked Bag 7, 6258 Manjimup, Australia; Julius Kühn-Institute (JKI), Institute for Breeding Research on Fruit Crops, Federal Research Centre for Cultivated Plants, Pillnitzer Platz 3a, 01326 Dresden, Germany; Julius Kühn-Institute (JKI), Institute for Breeding Research on Fruit Crops, Federal Research Centre for Cultivated Plants, Pillnitzer Platz 3a, 01326 Dresden, Germany; Department Molecular Plant Nutrition, Leibniz Institute of Plant Genetics and Crop Plant Research (IPK), Corrensstr. 3, 06466 Stadt Seeland, OT Gatersleben, Germany; Institute of Crop Science, Section of Crop Physiology of Specialty Crops (340f), University of Hohenheim, Emil-Wolff-Street 25, 70599 Stuttgart, Germany

**Keywords:** alternate bearing, apple, flower induction, phytohormone profiling, plant hormones

## Abstract

Apple (*Malus × domestica* Borkh.) cropping behavior, if not regulated, is often manifested by high yields of small-sized fruit in so called ON-years, which are usually followed by strongly reduced crop loads in OFF-years. Such cropping pattern is defined as biennial bearing and causes significant losses in apple production. The growth of apple fruit overlaps with the formation of flower buds, which remain dormant until the following spring. Earlier works proposed that some fruit-derived mobile compounds, as e.g., phytohormones, could suppress flower bud formation that thereby leads to biennial bearing. We addressed this hypothesis by analyzing 39 phytohormones in apple seeds, fruit flesh and by measuring phytohormone export from the fruits of the biennial bearing cultivar ‘Fuji’ and of the regular bearing cultivar ‘Gala’. Moreover, we analyzed the same compounds in bourse buds from fruiting (ON-trees) and non-fruiting (OFF-trees) spurs of both apple cultivars over the period of flower bud formation. Our results showed that apple fruit exported at least 14 phytohormones including indole-3-acetic acid and gibberellin A3; however, their influence on flower bud formation was inconclusive. A gibberellin-like compound, which was detected exclusively in bourse buds, was significantly more abundant in bourse buds from ON-trees compared with OFF-trees. Cultivar differences were marked by the accumulation of *trans*-zeatin-O-glucoside in bourse buds of ‘Gala’ ON-trees, whereas the levels of this compound in ‘Gala’ OFF were significantly lower and comparable to those in ‘Fuji’ ON- and OFF-trees. Particular phytohormones including five cytokinin forms as well as abscisic acid and its degradation products had higher levels in bourse buds from OFF-trees compared with ON-trees and were therefore proposed as potential promotors of flower bud initiation. The work discusses regulatory roles of phytohormones in flower bud formation in apple based on the novel and to date most comprehensive phytohormone profiles of apple fruit and buds.

## Introduction

Flower induction in plants is controlled by a complex gene regulatory network and is affected by multiple environmental and internal cues. In model organisms, such as *Arabidopsis thaliana*, this network is quite well characterized whereas in trees regulatory mechanisms of flowering remain poorly understood. In apple (*Malus × domestica* Borkh.), homologs of many important flowering time-regulating genes have been identified, and some of these have been functionally verified (Flachowsky et al. 2012, Kotoda et al. 2010, Tränkner et al. 2010, Kagaya et al. 2020). It is known that ambient temperature (Heide et al. 2020), carbohydrate supply (Wünsche and Ferguson 2010, Xing et al. 2015) and plant hormones (Krasniqi et al. 2013, Xing et al. 2015, Zhang et al. 2019) play an important role in flower development in apple. However, it is unclear, where the signal cascade leading to flower induction starts and what compound serves as a trigger for flower development in the newly formed buds (Goldschmidt and Sadka 2021).

Apple trees bloom during the springtime, however, flower bud formation occurs in the summer of the previous year, simultaneously with the fruit growth. Initially, every apple bud contains only leaf meristem, which under flower inductive conditions can also form flower organs. Flower bud formation begins with flower induction, during which the bud meristem registers one or more unknown inductive signals. At this stage, morphological changes in the buds are not yet detectable. This is followed by flower bud initiation with detectable broadening and dooming of the apical bud meristem. During this phase, the bud switches from a vegetative to a generative status (Buban and Faust 1982, Hanke 1981, Kofler et al. 2019). This process continues with the meristem differentiation into separate flower compartments (Foster et al. 2003). Once flower buds are formed, they remain dormant until the next spring, when the phenological sequence continues with budbreak and flowering.

Apple cropping behavior is often described as irregular, which is expressed by strongly reduced yields (OFF-years) after seasons when the trees were overloaded with fruit (ON-years). Such cropping behavior is called alternate, or biennial, bearing and leads to severe economic losses in fruit production (Monselise and Goldschmidt 1982, Wünsche 2019). Some apple cultivars, like ‘Gala’, are regular (non-biennial) bearing, whereas the others, like ‘Fuji’, are characterized with biennial cropping behavior. Many studies indicated that the presence of the fruit (Weinbaum et al. 2001) and, in particular, seeded fruit (Chan and Cain 1967) negatively affect flower bud development in apple. However, the physiological mechanism underlying the suppression of flower bud formation by the fruit is still largely unknown (Fulford 1966, Guitton et al. 2016).

To date, the leading hypotheses explaining the mechanism of such fruit-bud interactions are (i) the competition for carbohydrates between the simultaneously developing fruit and buds (Pawar and Rana 2019) and (ii) inhibition of flower bud initiation caused by signaling compounds, e.g., phytohormones, which are produced in the fruit (flesh or seeds) and which are subsequently transported to the adjacent buds (Dennis and Neilsen 1999). In this case, four criteria have to be met: the signaling compound (i) must have a negative effect on flower bud formation, (ii) has to be present in seeds and/or fruit flesh, (iii) has to be transported or diffused from the place of origin to the buds, (iv) has to be more abundant in the buds from high-cropping (ON) trees compared with the buds from non-cropping (OFF) trees or it has to be present exclusively in the buds from ON-trees.

The involvement of plant hormones in flower bud development in apple has been shown in many field studies. For example, foliar applications of the synthetic cytokinin 6-benzyladenine (6-BA) on apple trees increased flowering rate in the following spring (Li et al. 2016). In contrast, gibberellins 3 and 4 + 7 (GA3 and GA4 + 7) reduced the percentage of flowering spurs (McLaughlin and Greene 1991, Zhang et al. 2016). The applications of the synthetic auxin 1-naphthaleneacetic acid (NAA) in apple orchards either had a positive effect on return bloom in the following year (McArtney et al. 2013, Stopar 2010, Zabrkić et al. 2011) or did not significantly affect return bloom of the experimental trees (Guak et al. 2002). These examples show that some phytohormones or their synthetic analogs might determine whether a bud will become floral or not. For this reason, plant hormones are among the most promising candidates for the still unknown signaling substances, which either promote or inhibit flower bud formation.

Plant seeds produce phytohormones and employ them for embryo development and regulation of the seed dormancy status (Chen et al. 2018, Née et al. 2017). However, there is little known about the phytohormone composition in apple seeds. Previous studies mostly focused on the analysis of a few known auxins and gibberellins (GAs) in apple seeds postulating their importance for fruit development and proposing their potential influence on flower bud induction (Hoad 1978, Luckwill 1957). Tu (2000) studied GA composition in apple seeds of ‘Gala’ and ‘Fuji’ and reported 16 different GAs and 6 GA-like substances, which were detected in the seeds of these two apple cultivars. Besides GAs, apple seeds contain indole-3-acetic acid (IAA) that was detected in ‘Gala’ apple seeds by Devoghalaere et al. (2012). The authors also found IAA in the apple cortex, yet in much lesser amount. There is even less evidence on the presence of phytohormones in the buds of apple or closely related fruit trees, especially in connection to flower induction. In field experiments, Ito et al. (1999) stimulated flower development in lateral buds of Japanese Pear by shoot bending, a method known to promote flower bud formation in pome fruit (Lauri and Lespinasse 2001). The results of this study indicated that the lateral buds of bent shoots had lower concentrations of auxin (IAA) and (GA1 + 3 and GA4 + 7) in comparison to the vertical shoots, whereas the concentrations of abscisic acid (ABA) and cytokinins (*trans*-zeatin, tZ and *trans*-zeatin riboside, tZR) were significantly higher in lateral buds of bent shoots. Similar results were obtained by Zhang et al. (2015) who analyzed phytohormones in spur and terminal buds on bent shoots of ‘Fuji’ and ‘Gala’ apple cultivars. These results suggested that flower bud development can be stimulated by changing the hormonal balance in the buds of bent shoots.

The question whether plant hormones can diffuse from the fruit and thereafter reach adjacent bourse buds has been considerably less studied than plant hormones in apple buds and fruit. These compounds can be captured in a buffer solution, in which the harvested fruits are immersed with the pedicels. Such samples are known as apple fruit diffusates (Callejas and Bangerth 1998, Hoad 1978). In a similar way, plant hormones can be extracted from shoot apices, leaves and internodes, as it was shown by Grauslund (1972). Callejas and Bangerth (1998) were able to detect IAA in apple fruit diffusates and revealed that the quantity of diffusible IAA was influenced by the number of seeds (per fruit). The literature provides no more evidence on the translocation of the compounds from fruit to spurs or to the adjacent bourse buds. Hence, this topic still remains poorly researched and deserves more attention.

In order to understand the origin of biennial bearing in apple and to test the hypothesis whether particular phytohormones represent signaling substances, which are produced in the fruits and potentially inhibit flower bud formation, we conducted a comprehensive plant hormone profiling of apple fruit (seeds and fruit flesh), fruit diffusates and buds of two apple cultivars (‘Fuji’ and ‘Gala’) differing in their cropping behavior (biennial and non-biennial, respectively).

In our previous studies, we precisely determined the time of flower bud initiation in ‘Fuji’ and ‘Gala’ by using histological sectioning of bourse buds collected from fruiting spurs (ON-trees) and non-fruiting spurs (OFF-trees). Moreover, we studied transcriptome, proteome and metabolome of bourse buds collected from ON- and OFF-trees throughout 4 weeks prior to flower initiation (Kofler et al. 2019, Milyaev et al. 2021). These experiments revealed an upregulated plant hormone signal transduction pathway in the buds collected from ON-trees. Therefore, we used bud samples collected in the same field experiment in order to analyze 39 known phytohormones and to search for those, which differed between bourse buds collected from ON- and OFF-trees of ‘Fuji’ and ‘Gala’. The approach aimed to reveal phytohormones, which may be involved in flower bud formation (higher abundance in bourse buds from OFF-trees) or in pathways, which could suppress flower bud initiation (higher abundance in bourse buds from ON-trees, especially among the compounds, which were exported from the fruit). For the first time, we performed plant hormone profiling of apple seeds, apple fruit flesh as well as leaf and fruit diffusates and provided the data on 35 detected compounds.

## Materials and methods

### Field experiment and sampling

**Figure 1.**
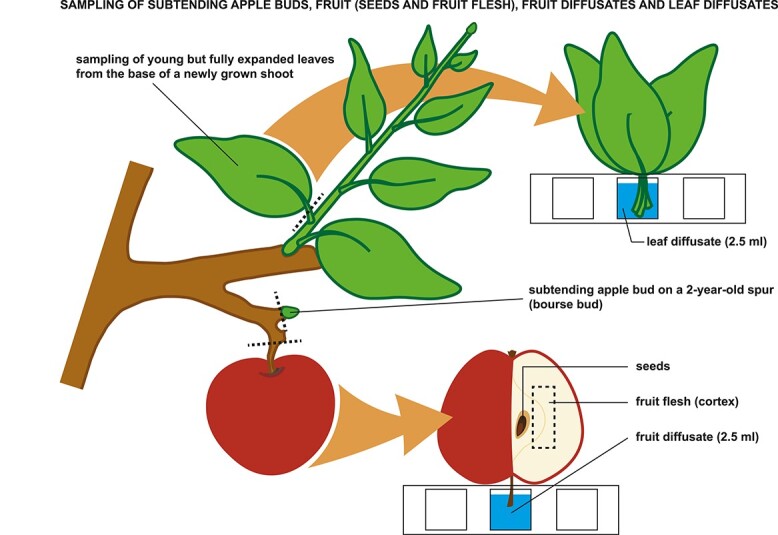
Sampling of subtending apple buds, fruit (fruit flesh and seeds), fruit diffusates and leaf diffusates.

**Figure 2.**
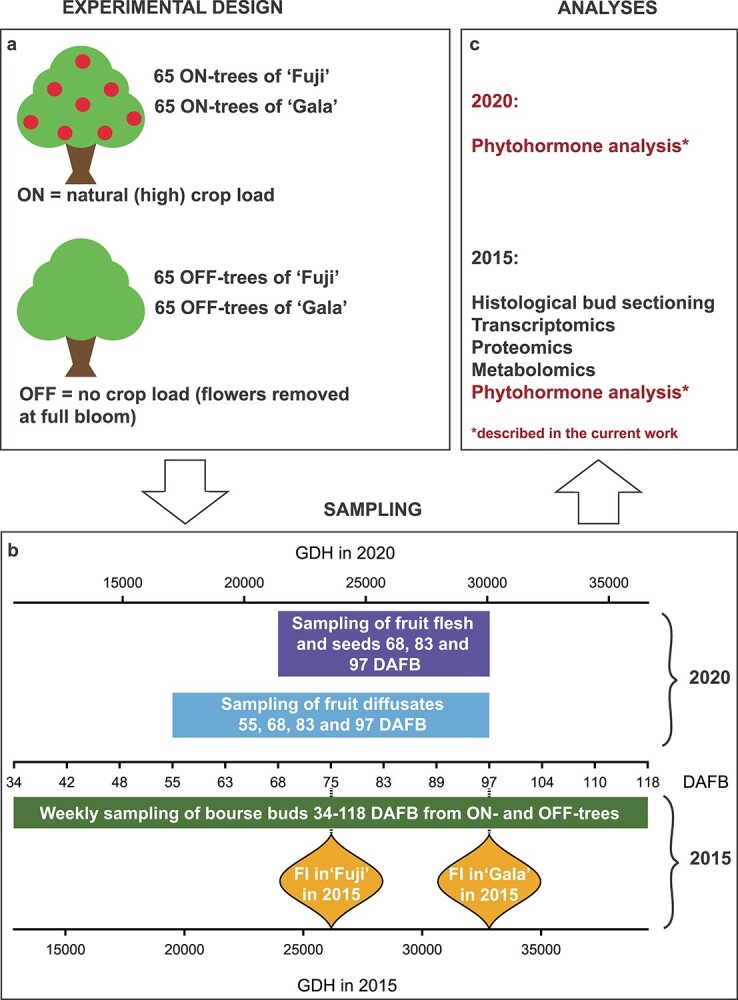
Scheme of experimental design (a), sampling (b) and analyses (c) described in the current work. Leaf diffusates were sampled 55 DAFB only. GDH (growing degree hours) were calculated according to Luedeling et al. (2009) from the day of budbreak (24 March in 2015 and 11 March in 2020 for both ‘Fuji’ and ‘Gala’). Flower bud initiation (FI) in ‘Fuji’ OFF occurred 75 DAFB (14 July, 25,650 GDH), in ‘Gala’ OFF and ‘Gala’ ON—97 DAFB (5 August, 32,946 GDH). No flower bud initiation in ‘Fuji’ ON-trees was detected.

Field experiment was conducted at the Competence Center for Fruit Cultivation at Lake Constance (KOB) near Ravensburg, Germany (47°46'2.89"N 9°33'21.21"E, altitude 490 m) on 7-year-old apple trees of two cultivars: ‘Fuji’ clone Raku-Raku (biennial bearing cultivar) and ‘Gala’ clone Galaxy (regular bearing cultivar) grafted on M.9 rootstock. The trees of each cultivar were allocated in two neighboring rows with spacing of 3 × 1 m (130 trees per cultivar), they were trained as tall spindles and were managed according to the standard for the region high-density orchard practices. At the stage of full bloom (30 April 2015), half of the experimental trees of each cultivar were completely thinned in order to bring the trees to the zero-cropping status (OFF-trees). The remaining trees were left without any flower or fruit thinning maintaining their natural crop load (ON-trees). Starting 34 days after full bloom (DAFB), subtending apple buds growing on 2-year-old spur wood (bourse buds) were sampled for the analyses (Figure 1). Sampling was performed with weekly interval from 34 to 118 DAFB (Figure 2). Each sampling week, a new set of eight apple trees per cultivar was sampled (four ON-trees and four OFF-trees). From each tree (=biological replicate), 55 bourse buds were collected. After the brown bud scales were removed, the buds were snap-frozen in liquid nitrogen and stored at −80 °C until required.

Fruit diffusates, fruit flesh and seeds for plant hormone analysis were sampled from ON-trees in 2020 during the period of flower bud development—at 55, 68, 83 and 97 DAFB for fruit diffusates and at 68, 83 and 97 DAFB for fruit flesh and seeds (Figure 2). For fruit diffusates, 32 fruit per cultivar (8 fruit per tree in 4 replicates) were collected at each out of four sampling time points. Immediately after harvest, the fruit were placed on the 24-well plates so that their pedicels (Figure 1) were immersed in a buffer solution, which was filled in the wells. Each well contained 2.5 ml of 0.1 M phosphate buffer at pH of 6.2 and 200 mM ethylenediaminetetraacetate in order to prevent phloem blockage. Apples on the plates were incubated at 20 °C for 20 h. After incubation, the plates were stored at −20 °C until analysis.

Identically to the fruit diffusates, leaf diffusates were prepared from young but fully expanded leaves collected 55 DAFB from the base of 1-year-old shoots. Leaves were collected in three biological replicates (tree = biological replicate) with each replicate consisting of three leaves put in one well with 2.5 ml buffer solution. Fruit flesh and seeds were collected in the orchard from four representative fruit per tree (=biological replicate) in four replicates. Each fruit was cut through in the transverse plane in order to collect seeds from the carpellary tissue and to sample fruit flesh tissue from the mesocarp (Figure 1). Seeds and fruit flesh were snap-frozen and stored at −80 °C until analysis.

### Analysis of phytohormones

#### Chemicals

Organic solvents methanol (MeOH) and acetonitrile (ACN) were purchased from Th. Geyer GmbH & Co. KG (Renningen, Germany). Formic acid (FA) was obtained from Biosolve Chimie (Dieuze, France). Deionized distilled water from the Milli-Q® Reference System, Merck (Darmstadt, Germany) was used for preparation of all mixtures and as mobile phase. The standards of phytohormones were purchased from OlChemim s.r.o. (Olomouc, Czech Republic).

#### Phytohormone extraction and quantification

For plant hormone analysis, 20 frozen buds from the mixture of 55 buds per biological replicate were taken and ground to powder using mortar and pestle cooled with liquid nitrogen. From each replicate, 30 ± 5 mg (fresh weight, FW) of bud powder was quickly weighted on the analytical scale in pre-cooled 2 ml safe-lock tubes (Eppendorf AG, Hamburg, Germany) and kept at −80 °C until extraction of plant hormones. Apple seeds and fruit flesh from each replicate were ground to powder in a cryogenic mixer mill (CryoMill, Retsch GmbH, Haan, Germany) at the frequency of 25 Hz using a 15-mm Ø ceria-stabilized zirconium oxide ball. From each replicate, 30 ± 5 mg (FW) of tissue powder was taken for the extraction of phytohormones and was prepared identically to apple bud samples. Fruit diffusates from each tree (eight diffusates) were mixed together and served as a replicate. From each mixture, 2.5 ml was taken for the analysis. For the analysis of leaf diffusates, three diffusates per cultivar were taken (each of them served as a replicate). All diffusates were freeze-dried until the dry white-yellowish phosphate powder from the buffer solution remained in the tubes (~30 mg DW). This powder was used for the extraction of plant hormones. Empty safe-lock tubes with extraction solvent were used as blanks. Prior to extraction, two 3-mm Ø ceria-stabilized zirconium oxide balls were placed into each safe-lock tube. Phytohormone extraction from plant tissue was performed according to Šimura et al. (2018) with some modifications. Briefly, 1 ml ice-cold 50% aqueous (v/v) ACN containing the internal standards was added to each tube for phytohormone extraction. Samples were homogenized in a MM 301 vibration mill (Retsch GmbH, Haan, Germany) operating at the frequency of 27 Hz for 5 min and afterward sonicated for 3 min at 4°C using a Sonorex ultrasonic bath (BANDELIN electronic GmbH, Berlin, Germany). Samples were subsequently placed in the Reax 32 overhead shaker (Heidolph Instruments GmbH, Schwabach, Germany) for at least 30 min. After 10 min centrifugation at 14,000 rpm and 4°C (CT 15 RE centrifuge, Himac, Japan), the supernatant was transferred to clean safe-lock tubes. All samples were purified using Oasis PRIME HLB RP (1 cc per 30 mg), polymer-based Solid Phase Extraction (SPE) cartridges (Waters Co., Milford, USA). After loading the supernatant, the flow-through fraction was collected in a clean tube. The cartridge was then rinsed with 1 ml of 30% (v/v) ACN, and this fraction was collected in the same tube as the flow-through fraction. After this single-step SPE, the samples were evaporated to dryness at 40 °C in a vacuum concentrator RVC 2-33 IR (Martin Christ GmbH, Osterode am Harz, Germany) and stored at −20 °C until analysis. For ultrahigh performance liquid chromatography - electrospray ionization tandem mass spectrometry (UHPLC-ESI-MS/MS) analysis, the samples were dissolved in 50 μl of 30% ACN (v/v) containing 0.1% FA and transferred to insert-equipped vials. The absolute quantification of all targeted phytohormones but GAs was performed by ultrahigh performance liquid chromatography - tandem mass spectrometry (UHPLC-MS/MS) as described in Eggert and von Wirén (2017). For the analysis of GAs, an LC-HRMS (liquid chromatography with high resolution mass spectrometer) method was used. Chromatographic separation was performed on a Vanquish™ UHPLC system from Thermo Fischer Scientific (San Jose, CA, USA). GAs baseline separation was achieved on a reversed phase Acquity UPLC® HSS T3 column (100 Å, 2.1 × 150 mm, 1.8 μm, Waters) using a gradient elution of A (Water, 0.1% FA) and B (MeOH, 0.1% FA) as follows: 0.0–0.3 min, 10% B; 0.3–0.7 min, 10–30% B; 0.7–2.0 min, 30–50% B; 2.0–4.0 min, 50–60% B; 4.0–8.0 min, 60% to 80% B; 8.0–9.5 min, 80–99% B; 9.5–10.4 min, 99% B. To preserve the integrity of the column, a guard column (130 Å, 2.1 × 5 mm, 1.8 μm, Waters) was also used. The column temperature was set at 45 °C and the flow rate at 0.3 ml/min. The injection volume was 5 μl. The UHPLC system was coupled to Q Exactive Plus Mass Spectrometer (San Jose, CA, USA) equipped with a Heated Electrospray (HESI) source operating in negative ion mode. Source values were set as follows: spray voltage 2.5 kV; capillary temperature 255 °C; S-lens RF level 40; aux gas heater temp 320 °C; sheath gas flow rate 47; aux gas flow rate 11. For spectra acquisition, a Full MS/dd-MS^2^ experiment was performed. The resolution in Full Scan was set as 70,000. For MS/MS experiments, the resolution was 17,500 and normalized collision energy was set at 40 V. MS data were acquired and processed by Trace Finder Software (v. 4.1, Thermo Scientific, San Jose, CA, USA). Twelve-point curve was prepared from standards mix solutions in the range of 0.5–1000 nM. In order to generate the calibration curve, the peak area on the extracted ion chromatogram of the deprotonated molecule ion [M-H]- was measured. A least-square linear regression was used to best fit the linearity curve. The identification of compounds found in extracts was based on retention time (RT), high-resolution m/z spectrum and isotope pattern with standards. Additionally, generated MS^2^ spectra were aligned with a custom spectral library for the confirmation of compound identification. Statistical differences between the cultivars (for seeds, fruit flesh and diffusates) and treatments (ON and OFF, for bourse buds) were determined with the *t*-test (at *P*-values < 0.01 and <0.05) that was performed separately for each sampling time point on Perseus 1.6.14.0 (Tyanova et al. 2016).

## Results

### Overview on phytohormones detected in apple buds, fruit and diffusates

**Figure 3.**
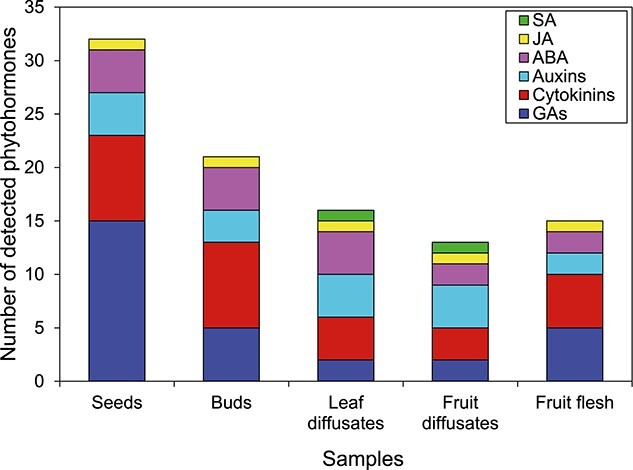
Number of phytohormones detected in surveyed samples and grouped by hormone classes.

Phytohormone profiling of all the plant tissues and diffusates was performed using a targeted approach with 39 reference compounds of the known plant hormones. These included cytokinins, auxins, gibberellic, jasmonic acid (JA), salicylic acid (SA), ABA and phaseic acids (PAs). With the applied analytical method, 31 phytohormones and 4 phytohormone-like compounds were detected in any sample type collected from the experimental apple trees, whereas seven compounds could not be detected in any of the samples (Table 1). The majority of phytohormones (32 compounds) were found in apple seeds, much less (15 compounds) were detected in apple fruit flesh, 14 and 16 plant hormones were identified in fruit and leaf diffusates, respectively (Figure 3). Among the analyzed samples, apple buds were characterized by the second highest number of the detected phytohormones (21 compounds). Some of the detected plant hormones could not be quantified as their concentration was below the lowest limit of quantification (LLOQ). The compounds referred to as ‘similar to…’ appeared as signals at the RT close to the RT expected for the corresponding standard compounds (Table 1). They were quantified as compounds they were similar to; however, they were not confirmed with internal standards and therefore could not be fully identified.

**Table 1:** Overview on the phytohormones detected in surveyed apple samples.

List of targeted compounds			Detected in				
No.	Class	Compound name, abbreviation	Seeds	Fruit flesh	Fruit diffusates	Leaf diffusates	Buds
1	CK	*Trans*-zeatin, **tZ**	+	+	+		+
2	CK	*Trans*-zeatin riboside, **tZR**	+	≤LLOQ		+	+
3	CK	*Trans*-zeatin-O-glucoside, **tZOG**	+				+
4	CK	*Trans*-zeatin riboside-O-glucoside, **tZROG**	+				+
5	CK	Dihydrozeatin, **DHZ**					
6	CK	Dihydrozeatin riboside, **DHZR**	+		≤LLOQ	≤LLOQ	+
7	CK	*Cis*-zeatin riboside, **cZR**	+	≤LLOQ	+	+	+
8	CK	*Cis*-zeatin, **cZ**					
9	CK	N6-isopentenyladenine, **iP**	+	≤LLOQ			+
10	CK	N6-isopentenyladenenosine, **iPR**	+	≤LLOQ	+	+	+
11	Auxin	2-oxoindole-3-acetic acid, **OxIAA**	+	+	+	≤LLOQ	+
12	Auxin	Indol-3-acetic acid, **IAA**	+	+	+	≤LLOQ	≤LLOQ
13	Auxin	IAA-glutamate, **IAA-Glu**	+		+	≤LLOQ	+
14	Auxin	IAA-aspartate, **IAA-Asp**	≤LLOQ		≤LLOQ	≤LLOQ	
15	Auxin	IAA-alanine, **IAA-Ala**					
16	JA	Jasmonic acid, **JA**	+	+	+	+	+
17	SA	Salicylic acid, **SA**			+	+	
18	ABA	Abscisic acid, **ABA**	+	+	+	≤LLOQ	+
19	ABA	ABA-glutamate, **ABA-Glu**	+	+	+	≤LLOQ	+
20	ABA	Dihydrophaseic acid, **DPA**	+			≤LLOQ	+
21	ABA	Phaseic acid, **PA**	+			≤LLOQ	+
22	GA	Gibberellin A1, **GA1**	+	≤LLOQ			
23	GA	Gibberellin A3, **GA3**	+	≤LLOQ	+		
24	GA	Gibberellin A4, **GA4**	+				
25	GA	Gibberellin A5, **GA5**					
26	GA	Gibberellin A6, **GA6**					
27	GA	Gibberellin A7, **GA7**	+				
28	GA	Gibberellin A8, **GA8**					
29	GA	Gibberellin A9, **GA9**	+				
30	GA	Gibberellin A12, **GA12**	+				+
31	GA	Gibberellin A15, **GA15**	+				
32	GA	Gibberellin A19, **GA19**	+	+	+	+	+
33	GA	Gibberellin A20, **GA20**	+				
34	GA	Gibberellin A24, **GA24**	+				
35	GA	Gibberellin A29, **GA29**					
36	GA	Gibberellin A44, **GA44**	≤LLOQ				
37	GA	Gibberellin A51, **GA51**	+				
38	GA	Gibberellin A53, **GA53**	+			+	+
39	GA	Similar to Gibberellin A3, **Similar to GA3**					+
40	GA	Similar to Gibberellin A5, **Similar to GA5**	+	≤LLOQ			
41	GA	Similar to Gibberellin A6, **Similar to GA6**	≤LLOQ	+			
42	GA	Similar to Gibberellin A44, **Similar to GA44**					+

‘Plus’ symbol means that the corresponding compounds were detected and quantified in the samples. ≤LLOQ—the corresponding compounds were detected in the samples but not quantified. Blank cells show that the corresponding compounds were not detected in the samples.

Comparison of plant hormone concentrations in seeds and fruit flesh indicated that almost all of the detected compounds were much less abundant in fruit flesh compared with those found in seeds. The exceptions were ABA-glutamate (ABA-Glu) and a GA-like compound similar to GA6. ABA-Glu was found at equal seasonal average concentrations in both fruit flesh and seeds, whereas the substance similar to GA6 was found at higher concentration in fruit flesh compared with seeds (Table 1, Tables S1 and S2 available as Supplementary data at *Tree Physiology* Online). This compound was detected neither in diffusates nor in bourse buds. Among all the sampled plant tissues, the biggest number of the analyzed GAs was identified in apple seeds. SA was detected exclusively in fruit diffusates and leaf diffusates.

### Fruit-derived mobile phytohormones and hormonal response of bourse buds on high-crop load

Following the hypothesis that possible hormonal inhibitors of flower bud initiation originate from the fruit, we analyzed phytohormones in apple seeds, fruit flesh and fruit diffusates. The results showed that some of the plant hormones, which were found in apple seeds and fruit flesh, were also exported from apple fruit. It should be mentioned that in the current work, the term ‘export’ of phytohormones refers to apple fruit diffusates and not to *in situ* transport of phytohormones. Considering the fact that all the quantified phytohormones detected in apple diffusates were more abundant in seeds compared with fruit flesh, we mostly focused on the compounds detected in the seeds in combination with those, which could move from apple fruit to the diffusate buffer solution. The exact concentrations of plant hormones found in apple seeds and fruit flesh are shown in Tables S1 and S2 available as Supplementary data at *Tree Physiology* Online. Apple diffusates contained 14 detectable phytohormones. The abundance of these compounds was also checked in bourse buds collected from ON-trees (mostly vegetative buds) and OFF-trees (mostly flower buds). Phytohormones, which were more abundant in bourse buds from ON-trees compared with those from OFF-trees (especially among the compounds that were found in apple diffusates), were expected to have potential inhibitory effect on flower bud initiation.

#### Cytokinins

In total, eight cytokinins were detected in the seeds of ‘Fuji’ and ‘Gala’. Seven of them—N6-isopentenyladenine (iP), N6-isopentenyladenenosine (iPR), tZ, tZR, dihydrozeatin riboside (DHZR), *trans*-zeatin-O-glucoside (tZOG) and *trans*-zeatin riboside-O-glucoside (tZROG)—were more abundant in ‘Fuji’ seeds compared with the seeds of ‘Gala’ during the whole sampling period (Table S1 available as Supplementary data at *Tree Physiology* Online) and showed statistical differences between ‘Fuji’ and ‘Gala’ in at least one of the sampling dates (68–97 DAFB). *Cis*-zeatin riboside (cZR) was also more abundant in the seeds of ‘Fuji’ compared with those of ‘Gala’ 68 DAFB and was below the detection limit in ‘Gala’ seeds 83 and 97 DAFB. Concentrations of cZR (only for ‘Fuji’), tZR and iPR in apple seeds notably increased over the period of 68–97 DAFB, whereas the abundance of iP, tZ, tZROG showed the opposite trend. The most prominent differences between ‘Fuji’ and ‘Gala’ seeds were observed for tZ. This physiologically most active cytokinin form showed was, depending on the sampling week, 6.3–8.3-fold more abundant in ‘Fuji’ seeds compared with ‘Gala’ seeds (Table S1 available as Supplementary data at *Tree Physiology* Online).

In apple diffusates, three cytokinin forms were detected, which included iPR, tZ and cZR (Figure S1b, f, j available as Supplementary data at *Tree Physiology* Online). Comparing the abundances of these cytokinins in bourse buds from ON- and OFF-trees, we observed that bourse buds of high-cropping trees had lower concentrations of these phytohormones in both studied apple cultivars (Figure S1c–d, g–h, k–l available as Supplementary data at *Tree Physiology* Online).

#### ABA and related compounds

ABA, ABA-Glu as well as some products of ABA catabolism—PA and dihydrophaseic acid (DPA)—were detected and quantified in apple seeds. ABA, ABA-Glu and DPA were more abundant in ‘Gala’ seeds compared with the seeds of ‘Fuji’ with significant differences in at least one of the sampling dates (68–97 DAFB). The concentration of all four compounds in the seeds of both apple cultivars increased during the sampling period (Table S1 available as Supplementary data at *Tree Physiology* Online).

**Figure 4.**
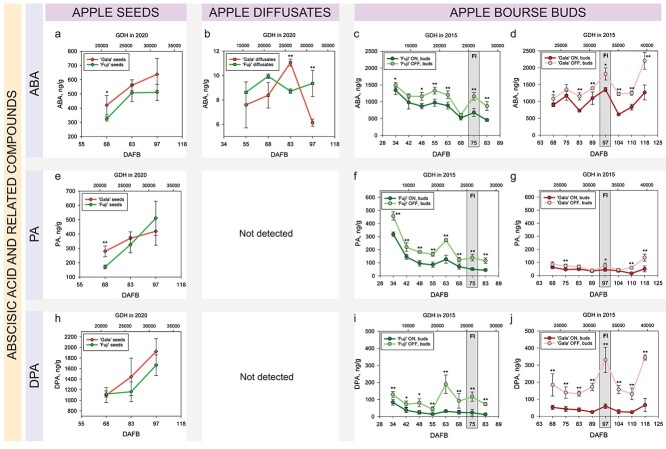
Concentrations of ABA (a–d), PA (e–g) and DPA (h–j) in seeds, fruit diffusates and bourse buds of ‘Fuji’ and ‘Gala’. Significant differences between cultivars and treatments are shown at *P* < 0.05 (^*^) and *P* < 0.01 (^*^^*^). Concentrations of phytohormones in seeds and buds are specified in ng/g of FW. Concentrations of phytohormones in apple diffusates are presented in ng/g of phosphate powder taken for the extraction of plant hormones after freeze-drying of apple diffusates (see Materials and methods). For the explanation of GDH and FI, please refer to the Figure 2.

ABA and ABA-Glu were found to be exported from apple fruit (Figure 4b and Figure S2b available as Supplementary data at *Tree Physiology* Online), whereas PA and DPA were not found in apple diffusates. Analysis of ABA and ABA-Glu in bourse buds revealed that ABA showed significantly higher levels in the buds from OFF-trees compared with those collected from ON-trees in ‘Fuji’ and ‘Gala’, whereas the opposite was true for ABA-Glu. Hence, the latter compound was detected in apple seeds, apple diffusates and in bourse buds being at the same time more abundant in the buds from high-cropping trees of both apple cultivars. However, comparison of seasonal average concentrations of ABA-Glu in apple seeds and in bourse buds revealed that this phytohormone was more abundant in bourse buds of both ON- and OFF-trees (41-fold and 27-fold, respectively) compared with seeds (Figure S2c and d available as Supplementary data at *Tree Physiology* Online). The abundance of ABA-Glu in bourse buds was fluctuating between 665–3,270 ng g^−1^ depending on cultivar, treatment and sampling date.

#### Gibberellic acids

In all the analyzed samples, we measured the concentrations of several GA precursors (GA12, GA15, GA24, GA9, GA7, GA53, GA19 and GA20) of three bioactive forms (GA1, GA3 and GA4) as well as of deactivated GA species (GA5, GA51) (Table 1). Among these GAs, the most abundant GA in apple seeds was GA4 with the concentration of 136–514 ng g^−1^ in ‘Gala’ and of 22–136 ng g^−1^ in ‘Fuji’. In contrast to cytokinins, this and another six detected GAs (including GAs A1, A3, A7, A9, A12, A24) showed clear tendency for higher abundance in the seeds of ‘Gala’ compared with the seeds of ‘Fuji’ with statistical significance in at least one of the sampling dates (68, 83, 97 DAFB). The over-time changes in concentrations of active GAs in apple seeds was not the same for all compounds. In particular, the levels of GA1, GA4 and GA7 in the seeds of ‘Fuji’ and ‘Gala’ decreased over the sampling period. In contrast, the concentrations of GA3 in the seeds of both apple cultivars increased over the sampling time (Table S1 available as Supplementary data at *Tree Physiology* Online).

**Figure 5.**
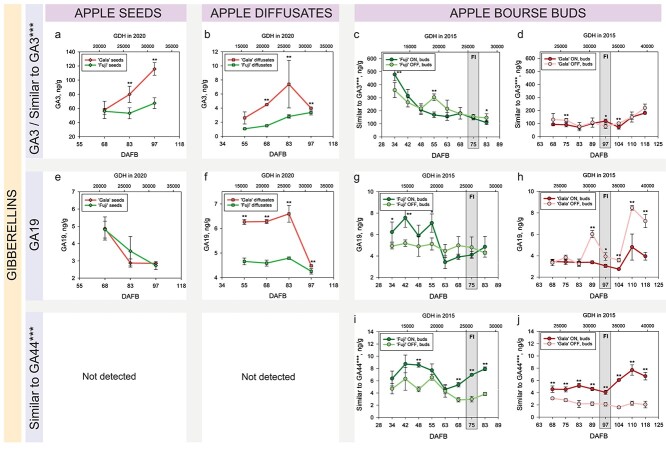
Concentrations of GAs (a–b, e–h) and relative abundances of a GA-like (^*^^*^^*^) compounds (c–d, i–j) in seeds, fruit diffusates and bourse buds of ‘Fuji’ and ‘Gala’. Significant differences between cultivars and treatments are shown at *P* < 0.05 (^*^) and *P* < 0.01 (^*^^*^). Concentrations of phytohormones in seeds and buds are specified in ng/g of FW. Concentrations of phytohormones in apple diffusates are presented in ng/g of phosphate powder taken for the extraction of plant hormones after freeze-drying of apple diffusates (see Materials and methods). For the explanation of GDH and FI, please refer to the Figure 2.

From the diversity of 12 GAs quantified in apple seeds, only two of them (GA3 and GA19) were found in apple diffusates. The concentrations of both gibberellic acids were significantly higher in the diffusates from the regular bearing cultivar ‘Gala’ compared with the diffusates from the biennial bearing cultivar ‘Fuji’ (Figure 5b and f). The data on GA19 in ‘Gala’ bourse buds showed that this phytohormone had significantly higher levels in the buds collected from OFF-trees compared with those from ON-trees starting 1 week prior to flower initiation and onwards (89–118 DAFB), whereas in ‘Fuji’ bourse buds, GA19 did not show clear differences between ON- and OFF-treatments during the sampling period. (Figure 5g and h). The bioactive GA3 was not detected in bourse buds. However, bourse buds contained another GA-like compound similar to GA3. This compound did not show clear differences between the buds collected from ON- and OFF-trees. Another three bioactive GAs (GA1, GA4 and GA7) were not found in bourse buds of ‘Fuji’ and ‘Gala’.

One more GA-like compound referred to as ‘similar to GA44’ was detected exclusively in bourse buds. Markedly, it showed clear treatment (ON and OFF) differences in both studied apple cultivars being significantly more abundant in ‘Fuji’ ON at 48, 68, 75 and 83 DAFB and in ‘Gala’ ON at all sampling dates (Figure 5i and j). Another two out of three known GAs detected in apple buds (GA12 and GA53) showed no clear statistical differences in their concentrations in bourse buds between ON- and OFF-trees (Figures S7 and S8 available as Supplementary data at *Tree Physiology* Online). Analysis of the known gibberellic acids did not clearly indicate any compound that was consistently higher or lower in bourse buds of either ON- or OFF-trees in both studied apple cultivars. However, the study indicated that bourse buds contained other GA-like compounds, the abundances of which were affected by ON- and OFF-treatments.

#### Auxins and JA

**Figure 6.**
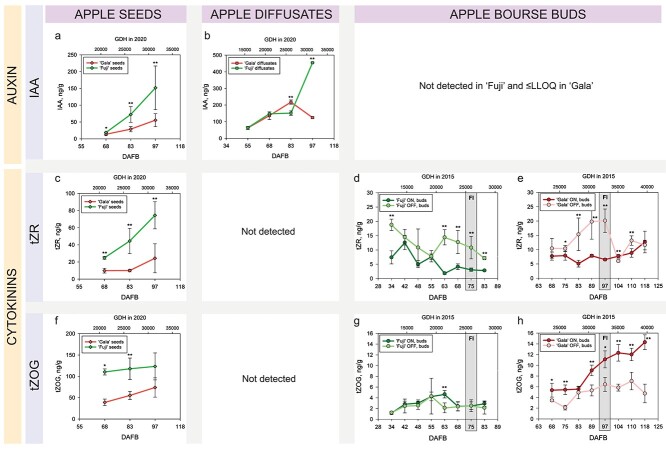
Concentrations of IAA (a, b), tZR (c–e) and tZOG (f–h) in seeds, fruit diffusates and bourse buds of ‘Fuji’ and ‘Gala’. Significant differences between cultivars and treatments are shown at *P* < 0.05 (^*^) and *P* < 0.01 (^*^^*^). Concentrations of phytohormones in seeds and buds are specified in ng/g of FW. Concentrations of phytohormones in apple diffusates are presented in ng/g of phosphate powder taken for the extraction of plant hormones after freeze-drying of apple diffusates (see Materials and methods). For the explanation of GDH and FI, please refer to the Figure 2.

Similar to cytokinins, all three auxin forms (IAA; IAA-Glutamate, IAA-Glu; 2-oxoindole-3-acetic acid, OxIAA) and JA had higher levels in ‘Fuji’ seeds compared with the seeds of ‘Gala’ with statistical differences in at least one of the sampling dates (Figure 6a, Figures S2e and S3a, e available as Supplementary data at *Tree Physiology* Online). The concentration of free auxin (IAA) in the seeds of both studied apple cultivars increased during the period of sampling. In ‘Fuji’ seeds, the abundance of IAA was 1.4-, 2.5- and 2.7-fold higher than that in ‘Gala’ seeds at 68, 83 and 97 DAFB, respectively (Figure 6a). All these differences were statistically significant. The time-series abundance patterns of IAA-Glu in the seeds of ‘Fuji’ and ‘Gala’ were cultivar-dependent and inversely proportional to those of OxIAA. The abundance of JA in apple seeds had no significant changes over the time of seed sampling (68, 83, 97 DAFB).

All four phytohormones were also detected in apple diffusates; however, three of them including IAA-Glu, OxIAA and JA did not show clear differences between bourse buds collected from ON- and OFF-trees (Figure S2f–h, Figure S3b–d and f–h available as Supplementary data at *Tree Physiology* Online). Among all the phytohormones detected in apple diffusates, the amounts of IAA exported from the fruit were the highest (Figure 6b) followed by much lower concentrations of ABA-Glu and JA. The levels of IAA in bourse buds were, however, below the quantification limit in ‘Gala’ and were not detectable in ‘Fuji’. It therefore indicates that the analysis did not provide sufficient information on whether IAA content in apple buds was affected by high-crop load.

### Hormonal response of apple bourse buds on zero crop load

In order to understand whether any of the phytohormones detected in apple bourse buds could promote flower initiation, we analyzed abundance patterns of these compounds on a time-series scale (34–83 DAFB for ‘Fuji’ and 68–118 DAFB for ‘Gala’) that included the known time points of flower bud initiation for ‘Fuji’ (75 DAFB) and ‘Gala’ (97 DAFB). Plant hormones, which showed higher abundances in the buds from OFF-trees (mostly flower buds) compared with ON-trees (mostly vegetative buds) prior to flower bud initiation, were expected to have promotive effect on the development of primordial flower organs in apple bourse buds.

#### Cytokinins

Among eight cytokinin-related compounds detected in bourse buds of ‘Fuji’ and ‘Gala’, the abundance of the cytokinin precursor iPR was the highest. Its concentration in bourse buds was fluctuating between 4 and 25 ng g^−1^ in ‘Fuji’ and between 4 and 75 ng g^−1^ in ‘Gala’ depending on treatment and sampling date (Figure S1c–d available as Supplementary data at *Tree Physiology* Online). The abundance of iPR in bourse buds was followed by tZR with the concentration range of 2–19 ng g^−1^ in ‘Fuji’ and of 5–20 ng g^−1^ in ‘Gala’. Comparison of cytokinin levels in the buds from ON- and OFF-trees revealed a clear tendency of higher abundances of some cytokinins (tZ, tZR, cZR and DHZR) and cytokinin precursors (iP and iPR) in the buds from non-cropping trees in both apple cultivars (Figure 6d–e, Figure S1c–d, g–h, k–l, Figures S4 and S6 available as Supplementary data at *Tree Physiology* Online). Specifically, cytokinins tZR, cZR and DHZR were significantly more abundant in the buds collected from OFF-trees at several sampling time points (Figure 6d–e, Figure S1k–l, Figure S6). It is noteworthy that the concentration of tZR in the buds from OFF-trees markedly increased 2 weeks prior to flower initiation (63 and 68 DAFB in ‘Fuji’, 83 and 89 DAFB in ‘Gala’) in both apple cultivars (Figure 6d–e). Higher abundance of tZ in flower buds was less pronounced with significant differences between ON- and OFF-trees only 63 DAFB in ‘Fuji’ and 97 DAFB in ‘Gala’. The abundance pattern of tZOG and tZROG differed from other cytokinins. Both of them were more abundant in the buds collected from ON-trees of the regular bearing cultivar ‘Gala’. In particular, the abundance of tZOG in ‘Gala’ bourse buds collected from ON-trees was depending on the sampling week, 1.1–9.6-fold higher compared with the buds sampled from OFF-trees (Figure 6g–h).

#### ABA and related compounds

ABA had significantly higher levels in the buds collected from OFF-trees compared with ON-trees of both cultivars almost over the whole sampling period (except for 42 and 68 DAFB in ‘Fuji’ and 75 DAFB in ‘Gala’). The abundance of ABA in bourse buds laid between 457 and 1,492 ng g^−1^ in ‘Fuji’ and between 614 and 2,206 ng g^−1^ in ‘Gala’ depending on sampling week and treatment (Figure 4c–d). In the phytohormone profile of bourse buds, two products of ABA catabolism (PA and DPA) were also detected. Both compounds were more abundant in the buds from OFF-trees and showed similarities to the seasonal abundance pattern of ABA (especially in ‘Gala’). DPA levels in bourse buds collected from ON- and OFF-trees differed significantly between the treatments in both cultivars throughout the whole sampling period, whereas PA showed such differences only in ‘Fuji’ (Figure 4f–g, i–j).

Abundance patterns of the phytohormones detected in ‘Gala’ buds showed that the reduction of ABA concentration (such as at 83 and 104 DAFB) coincided with the increasing concentration of GA19 and cytokinins iP, iPR, cZR and tZ 1 week later (89 and 110 DAFB). Such events, however, were not observed in the buds collected from ‘Fuji’ trees.

### Phytohormones exported from apple leaves

Among 16 phytohormones detected in leaf diffusates, only seven and two compounds were above the LLOQ in ‘Fuji’ and ‘Gala’, respectively. SA and GA19 were quantified in both ‘Fuji’ and ‘Gala’ leaf diffusates, whereas GA53, JA, iPR, tZR and cZR could only be quantified in the leaf diffusates of ‘Fuji’. The analysis showed that SA was the most abundant in leaf diffusates followed by GA53 (found only in ‘Fuji’) (Figure S9). Since no comprehensive cultivar comparisons within the phytohormones detected in leaf diffusates appeared to be possible, the measurements in diffusates rather serve as a reference for the identified compounds, which exported from apple leaves 55 DAFB.

## Discussion

In this work we attempted to identify phytohormones, which determine the fate of apple bourse bud meristem and hence play a role in flower bud induction in apple. For this purpose, we searched for those plant hormones, which are synthesized in the fruit and which are contemporaneously exported from there as mobile compounds. Such phytohormones are most likely to reach the adjacent bourse buds where some of those compounds may inhibit flower bud initiation and, as a result, trigger biennial bearing in apple. At the same time, we searched for phytohormones with potentially promoting effects on flower bud initiation. Using state-of-the-art methods of mass spectrometry, we analyzed 39 phytohormones in apple seeds, fruit flesh, bourse buds, leaf diffusates and fruit diffusates and presented comprehensive phytohormone profiles of each tissue together with the overview on the compounds exported from fruit and leaves.

Analysis of phytohormones revealed that at least 14 compounds could be exported from apple fruit. Comparing the abundances of phytohormones quantified in both apple seeds and fruit diffusates on a time-series scale (68–97 DAFB) showed that the temporal abundance patterns of individual compounds in seeds were not identical to those observed in apple diffusates. The exceptions were JA, OxIAA and to some extend GA3. This demonstrated that for the compounds reported in this study, the temporal concentration changes of phytohormones in apple seeds did not have a direct connection with their amounts in apple diffusates. Moreover, the abundances of phytohormones detected in apple seeds did not correlate with their amounts found in apple diffusates.

**Figure 7.**
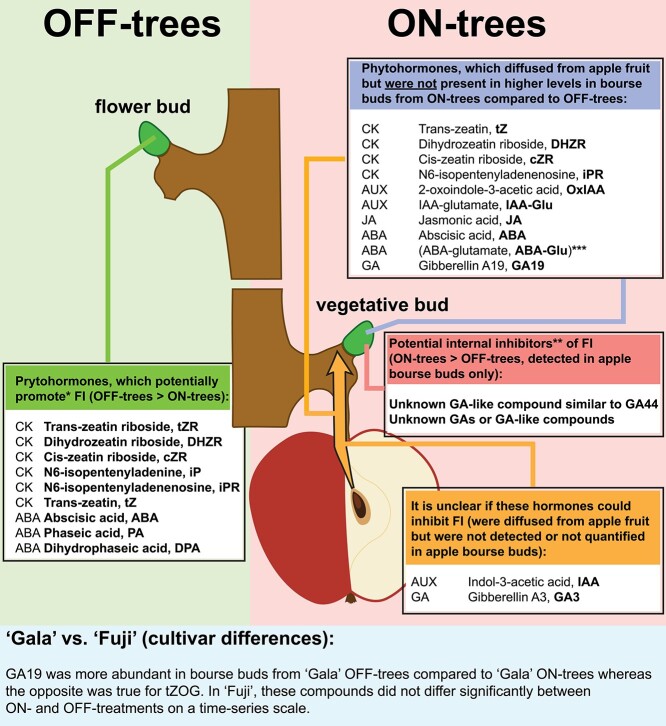
Summary on phytohormones with potentially regulatory roles in flower bud formation in apple. ^*^Potential promotors of flower bud initiation are proposed based on significantly higher abundance of the corresponding compounds in bourse buds from OFF-trees compared with bourse buds from ON-trees in any of the sampling time points during 4 weeks prior to and at the time of flower bud initiation in ‘Fuji’ and ‘Gala’. The proposed compounds were also characterized by clear trend of higher abundance in bourse buds from OFF-trees during 4 weeks prior to flower bud initiation. ^*^^*^Potential inhibitors of flower bud initiation are proposed based on significantly higher abundance of the corresponding compounds in bourse buds from ON-trees compared with bourse buds from OFF-trees in any of the sampling time points during 4 weeks prior to and at the time of flower bud initiation in ‘Fuji’ and ‘Gala’. The proposed compounds were also characterized by clear trend of higher abundance in bourse buds from ON-trees during 4 weeks prior to flower bud initiation. ^*^^*^^*^The abundance of ABA-Glu in bourse buds collected from ON-trees was higher compared with bourse buds from OFF-trees of both apple cultivars. However, the accumulation of ABA-Glu in bourse buds from ON-trees is likely to be connected with the ABA inactivation through the conjugation of ABA with glutamate. Since this conjugated ABA form is not known to be physiologically active, the authors did not consider ABA-Glu as a potential inhibitor of flower initiation.

Phytohormone profiles indicated that 11 compounds satisfied three conditions of the hypothesis describing mobile signals with potentially inhibiting effect on flower initiation: they were identified in apple seeds, they were exported from the fruit and they were also detected in apple bourse buds. These compounds included tZ, DHZR, cZR, iPR, IAA, OxIAA, IAA-Glu, JA, ABA, ABA-Glu and GA19 (Figure 7). However, 10 of them were rejected by other criteria of the hypothesis. Some of these phytohormones were more abundant in the buds from OFF-trees compared with ON-trees (tZ, DHZR, cZR, iPR, ABA, GA19), some did not show clear differences between ON- and OFF-treatments (IAA-Glu, OxIAA, JA) and one was present in bourse buds of both, ON- and OFF-trees, at much higher concentrations compared with seeds (ABA-Glu).

The remaining compound, IAA, which was found in apple diffusates, was already proven to be synthesized in apple fruit and to be exported from there as a mobile compound (Callejas and Bangerth 1998). Our work confirmed these findings and revealed that the main source of IAA in apple fruit is the seeds. Among all the compounds detected in apple diffusates, the amounts of IAA were the highest. In bourse buds, however, IAA was either below the LLOQ (in ‘Gala’) or was not detected in the bud samples (in ‘Fuji’). A possible reason for this might be poor extractability of IAA from lignin-rich tissues, including apple buds, due to stronger ‘matrix effects’ (Hashiguchi et al. 2021). Comparison of peak areas of IAA MS-signals in ‘Gala’ bourse buds showed no clear differences in IAA abundances between ON- and OFF-trees. In previous studies, field trials using synthetic auxin analog NAA for the treatment of apple trees did not support the theory of inhibitory effect of auxins on flower bud initiation. Spraying of apple trees with NAA did not reveal negative effects of this compound on return bloom (Guak et al. 2002). In some cases, NAA was even shown to be promotive for the next-year flowering (McArtney et al. 2013, Zabrkić et al. 2011). Even though the question whether IAA inhibits flower bud initiation in apple still remains open, the involvement of elevated auxin production and its export from fruits in the inhibition of flower bud formation cannot be excluded.

A study on auxin–GA interactions in pea stem segments cultivated *in vitro* demonstrated that the addition of IAA to growth media stimulated GA biosynthesis in the explants (O’Neill and Ross 2002). This observation raises the question whether IAA exported from the fruit could induce GA biosynthesis in the adjacent apple buds that in turn inhibits flower bud initiation. Therefore, interactions between different auxin and GA forms in apple buds or the crosstalk between auxin and other signaling compounds may merit further investigation.

Using the described analytical approach, we detected four bioactive GAs (GA1, GA3, GA4 and GA7) in apple seeds, two in fruit flesh (GA1 and GA3), one in apple diffusates (GA3) and none of them in bourse buds. The amount of GA3, which was exported from apple fruit, was significantly higher in the regular bearing cultivar ‘Gala’ compared with the biennial bearing cultivar ‘Fuji’ 68, 83 and 97 DAFB. All these results, however, do not provide clear evidence whether GA3 could be the candidate for a fruit-derived signal, which potentially inhibits flower initiation in apple.

Another GA that was exported from apple fruit was GA19. This compound is known to be a precursor of some active GAs, such as GA1 and GA3 (He et al. 2020). Cultivar comparisons showed that, as in the case with GA3, ‘Gala’ fruit exported larger amounts of GA19 compared with ‘Fuji’. At the same time, the amounts of GA19 in ‘Gala’ bourse buds from OFF-trees was significantly higher compared with the buds from ON-trees, whereas there were no clear differences between the ON- and OFF-treatments in ‘Fuji’. The increased concentration of GA19 in bourse buds of non-cropping ‘Gala’ trees did not, however, find any biological explanation and represents one of the few examples of cultivar differences between ‘Fuji’ and ‘Gala’ shown in the current work (Figure 7).

Among all the detected GAs, a GA-like compound similar to GA44 was significantly more abundant in the buds from ON-trees compared with OFF-trees suggesting that the potentially inhibiting effect of GAs on flower bud initiation could also be achieved through some unknown active GAs. During the analyses of MS-signals, we detected some additional peaks of GA-like compounds in bourse buds from ON-trees, whereas these signals were not present in bourse buds from OFF-trees. These compounds had fragmentation patterns typical to GAs; however, they could not be identified neither with chemical databases nor with the available standard compounds. This indicates that the spectrum of gibberellic acids in apple is not limited by the already discovered substances.

Research of the past decade demonstrated that the response of flowering-regulating genes on GAs and GA inhibitors may be different or even opposite in model plants and in fruit trees. In Arabidopsis, GAs were shown to be involved in flower development by promoting transcriptional activation of the flowering time genes *FLOWERING LOCUS T (FT)*, *SUPPRESSOR OF OVEREXPRESSION OF CONSTANS1 (SOC1)* and *LEAFY (LFY)*, as reviewed by Bao et al. (2020). At the same time, enhanced expression of *LFY* induces GA catabolism and leads to the reduction of GA levels in the inflorescence of Arabidopsis, suggesting that in this species GAs are not required during the whole phase of flower development (Yamaguchi et al. 2014). Foliar applications of paclobutrazol (GA inhibitor) reduced *FT* expression in wild-type Arabidopsis (Porri et al. 2012) as well as in the early flowering Arabidopsis mutant *arp6* and led to suppressed flowering of the latter (Galvão et al. 2015). In contrast, the opposite effects of sprayings with GAs and paclobutrazol were observed in fruit trees. For example, in mango (*Mangifera indica* L.), application with GA3 repressed flowering of the treated shoots and inhibited *FT* expression in the leaves (Nakagawa et al. 2012). In apple, foliar applications with GA4 + 7 increased the expression of flowering repressor *TERMINAL FLOWER 1-1 (TFL1-1)* in shoot apices and inhibited flower bud formation (Elsysy and Hirst 2019, Zhang et al. 2019). Contrary to the synthetic GAs, paclobutrazol applications positively affected return bloom in orange and in apple and increased the expression of *FT* gene in orange leaves (Muñoz-Fambuena et al. 2012) and in apple buds (Zhang et al. 2016). These results support the hypothesis that particular gibberellic acids may inhibit flower bud initiation in fruit trees (Figure 7). In the previous studies with ‘Golden Delicious’, Belhassine et al. (2019) detected 14 known GAs in terminal buds collected from ON- and OFF-trees at 58 DAFB. These included two biologically active GA forms, one of which (GA4) had similar concentrations in both treatments, whereas another active GA (GA1) was more abundant in the buds from OFF-trees. Therefore, the analysis of other potentially active GAs and GA-like compounds in apple buds could help to obtain a broader picture of other possible candidate suppressors of flower bud initiation.

Besides the molecules with potentially inhibitory effect on flower bud formation, we also targeted phytohormones, which could promote flower bud initiation. Such candidates encompass compounds, which were more abundant in bourse buds from OFF-trees compared with those from ON-trees, especially prior to flower initiation. These phytohormones included cytokinin precursors iP and iPR, cytokinins tZ, tZR, cZR and DHZR as well as ABA and its products of catabolism (PA and DPA). Leaf diffusates also contained some of these compounds, including iPR, tZR, cZR and DHZR. Future works might therefore reveal whether any of these or any other phytohormones are exported from leaves to buds and thereby stimulate flower bud development or whether the buds are capable of synthesizing of flower-inductive signal(s) by themselves.

A promotive role of cytokinins in flower bud development is indicated from studies with model plants (Wybouw and De Rybel 2019). In Arabidopsis, synthetic cytokinin 6-BA was proven to stimulate the expression of *SOC1* and *TWIN SISTER OF FT (TSF)*, which affect floral meristem identity genes that in turn initiate floral transition of the shoot apical meristem (D’Aloia et al. 2011, Lee and Lee 2010). Analysis of the shoot apical meristem of Arabidopsis for the concentrations of zeatin and iP under flower-inducing conditions (long days) showed increased levels of both cytokinins compared with plants under non-inductive short days (Corbesier et al. 2003). Even though there are many more known cytokinins in Arabidopsis (Sakakibara 2006), not every compound has been tested for its involvement in flower induction. The involvement of *SOC1* in flowering control has also been proven in apple (Goldschmidt and Sadka 2021). However, it is still unclear if cytokinins promote flower development in apple through the same regulatory pathways as those discovered in Arabidopsis. The evidence that certain cytokinins can promote flower bud development in apple is illustrated in the studies, in which apple trees were sprayed with 6-BA that increased flowering rate of the trees in the subsequent spring (Li et al. 2016). Our results showed that many of the analyzed cytokinins were more abundant in bourse buds from non-cropping (OFF) trees compared with the buds from high-cropping (ON) trees. In order to identify particular cytokinins, which may be involved in flower bud development in apple, we focused on 10 cytokinin forms known in plants. The results showed that cytokinin precursors and some of the corresponding active forms, i.e., iP, iPR, tZ, cZR, tZR and DHZR were more abundant in flower buds in both apple cultivars, ‘Fuji’ and ‘Gala’ (Figure 7). In terms of temporal coincidence, the abundance of tZR in apple buds appeared to correspond with flower bud formation as its concentration in flower buds increased 2 weeks prior to flower initiation. This was observed in ‘Gala’ and ‘Fuji’ despite the differences in timing of flower bud initiation (in DAFB) in these two cultivars. Among the cytokinins detected in bourse buds, one compound (tZOG) showed substantial cultivar differences (Figure 7). In ‘Gala’, the concentration of tZOG in bourse buds from ON-trees considerably increased during the sampling period, whereas it remained stable in the buds from OFF-trees. The levels of tZOG in ‘Fuji’ bourse buds collected from both ON- and OFF-trees were comparable to those in ‘Gala’ OFF and did not significantly differ from each other. If the involvement of cytokinins in flower bud formation will be confirmed, the accumulation of tZOG in the buds of high-cropping ‘Gala’ trees may provide an explanation why some cultivars (including ‘Gala’) are less prone to biennial bearing and still can develop some amount of flower buds after the years with high-crop load.

Using the samples from the same field experiment, we previously analyzed the transcriptome and the proteome of the buds collected from ON- and OFF-trees. In the buds from OFF-trees of ‘Fuji’ and ‘Gala’, enrichment analysis indicated pathways attributed to cell division (such as DNA replication, purine metabolism, pyrimidine metabolism etc.), which are associated with physiological processes normally promoted by cytokinins (Milyaev et al. 2021). These enriched metabolic pathways indicate probable apple bud responses to elevated cytokinin activities with enhanced cell division that may lead to the formation new bud tissues.

Regarding the involvement of ABA in flowering time control, there are controversial studies reporting positive or negative effects of ABA on flower bud development in plants (Shu et al. 2018). ABA mediates drought stress responses in plants and can simultaneously accelerate flower transition by activating the expression of the florigens *FT* and *TSF*, as it has been shown in Arabidopsis (Riboni et al. 2013). On the other hand, ABA also inhibited flower development in Arabidopsis through its repressive effect on the expression of *SOC1* (Riboni et al. 2016). Our results demonstrated that ABA was more abundant in bourse buds from OFF-trees compared with bourse buds from ON-trees in both studied cultivars, indicating potential involvement of ABA in flower bud development in apple (Figure 7). Higher abundances of ABA, tZ and tZR in apple bourse buds from OFF-trees compared with those from ON-trees confirmed previous findings of Zuo et al. (2018) as well as the results on comparative phytohormone analysis in terminal and spur buds of bent shoots, with higher proportion of flower buds, compared with vertical shoots, with lower proportion of flower buds (Zhang et al. 2015). However, the involvement of ABA in flower bud induction or initiation as well as its effect on flowering genes in apple still remains to be determined.

PA and DPA, the known catabolites of ABA in plants (Chen et al. 2020), were also found to have higher abundances in bourse buds collected from OFF-trees compared with the buds from ON-trees. Despite being a degradation product of ABA, PA has been described as a plant hormone, which can enhance drought tolerance of plants and as a compound, which is able to activate a subset of ABA receptors (Rodriguez 2016), supporting its role as a signaling molecule. There is, however, no evidence on PA and DPA in relation to flower bud development, neither in model plants nor in fruit trees. Higher accumulation of either compound in bourse buds from OFF-trees was probably connected to higher ABA levels in them and hence to more intense ABA catabolism in the buds. However, the precise role of these compounds in apple buds remains to be discovered.

Flower induction and flower initiation in plants are the processes of great physiological complexity. Phytohormone profiles of apple fruit and buds provided an overview on the possible signaling compounds, which could be involved in the regulation of flower bud initiation in apple. Once the regulatory roles of particular phytohormones in flower bud formation are fully elucidated and validated by the additional experiments, it would be necessary to study the relative levels of promotors and inhibitors in bourse buds. Such approach together with any possible interconnections between different plant hormones and other constituents of the flower induction mechanism would provide a more nuanced view on the sequence of physiological events in apple buds leading to flower bud formation.

## Concluding remarks

The goal of this study was a comprehensive analysis of phytohormones in apple fruit and buds in order to reveal signaling compounds, which could inhibit or promote flower bud initiation in apple. Among all the tested compounds, IAA showed the highest abundance in apple diffusates; however, it could not be quantified in apple bourse buds. Bioactive GA3 was also exported from apple fruit but was not detected in bourse buds. Therefore, the question of inhibitory effects of IAA and GA3 on flower bud formation in apple still remains open. Besides that, we detected some unknown GA-like compound, which could be considered as potential inhibitor of flower bud initiation. Moreover, our results showed that other GA-like compounds were present in the buds from ON-trees, whereas they were absent in buds from OFF-trees.

Apart from that, we identified several phytohormones with potentially promotive effect on flower bud initiation in apple. Based on our findings, these compounds include cytokinins iP, iPR, tZ, tZR, cZR and DHZR as well as ABA, PA and DPA. The data showed that the abundance of tZR in bourse buds collected from OFF-trees increased 2 weeks prior to flower bud initiation in both cultivars, suggesting its involvement in the commencement of flower bud development in apple. The abundance tZOG in ‘Gala’ bourse buds from ON-trees significantly increased during the season indicating that higher levels of tZOG in bourse buds may be connected with the enhanced flower bud formation in regular bearing apple cultivars, such as ‘Gala’.

The phytohormone profiles of apple fruit and buds covered only a small fraction of all the existing signaling plant molecules but showed a significant portion of the known substances with phytohormonal activity. Besides those compounds, flower bud development could be influenced by other plant hormones, such as strigolactones, brassinosteroids, ethylene and melatonin. The evidence on the involvement of these phytohormones in flower bud development in both model plants and fruit trees is still scarce. Analysis of those compounds could further improve the current state of knowledge about flower bud induction in apple, especially in connection to biennial bearing.

## Supplementary Material

Suppl_fig_1-3_tpac083

Suppl_fig_4-8_tpac083

Suppl_fig_9_tpac083

Supplementary_tables_1_and_2_tpac083
