# Supplementary material for: Profiling of phytohormones in apple fruit and buds regarding their role as potential regulators of flower bud formation
Source: Tree Physiol. 2022 Aug 11;42(11):2319–35. doi: 10.1093/treephys/tpac083 (PMC9912367; doi:10.1093/treephys/tpac083)
Supplement: Suppl_fig_1-3_tpac083 [file suppl_fig_1-3_tpac083.pdf]

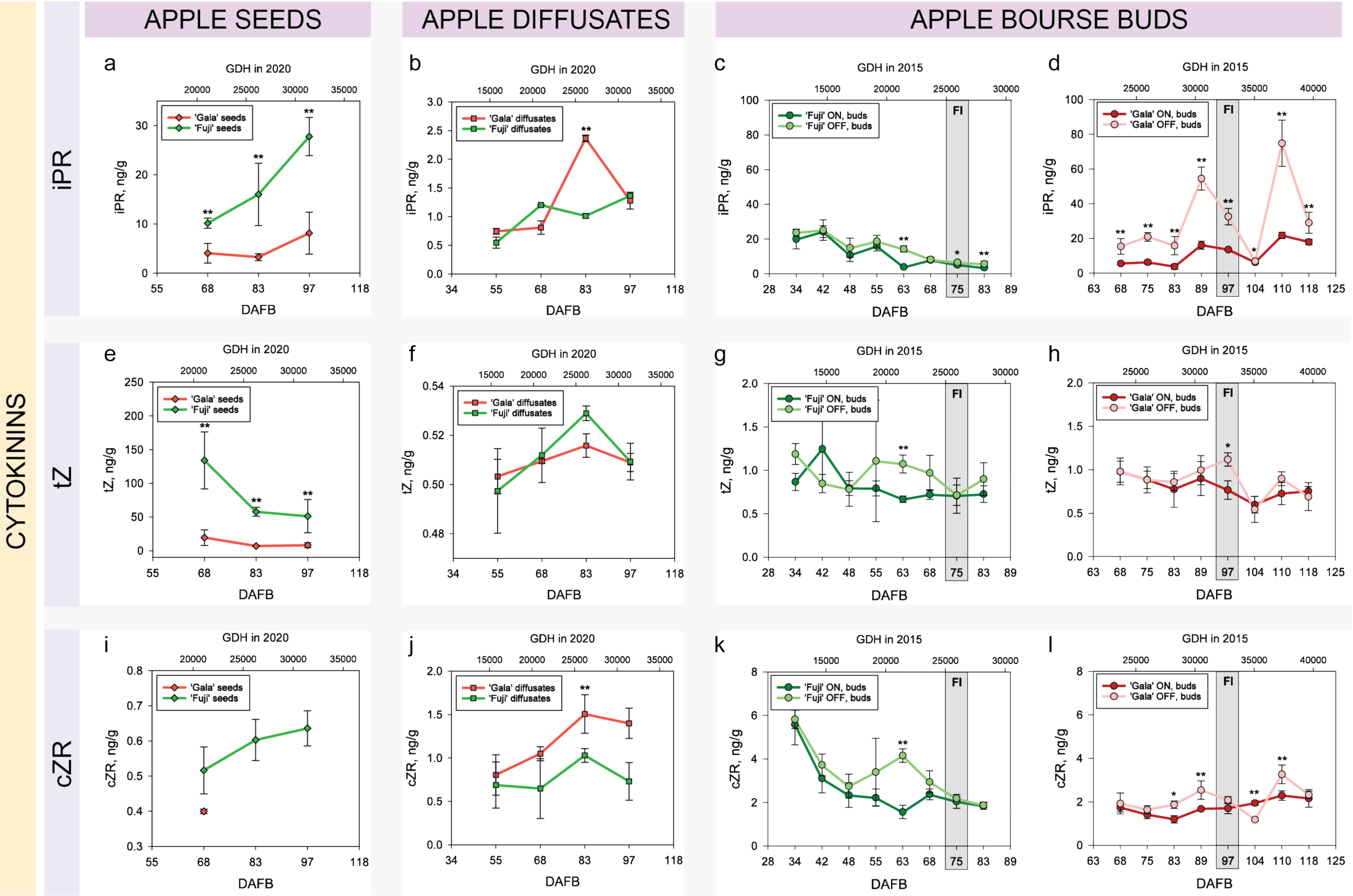

Supplementary figure 1. Concentration of iPR (a-d), tZ (e-h) and cZR (i-l) in seeds, fruit diffusates and bourse buds of ‘Fuji’ and ‘Gala’.

Significant differences between cultivars and treatments are shown at $p<0.05$ (*) and $p<0.01$ (**). Concentrations of phytohormones in seeds and buds is specified in ng/g of FW. Concentrations of phytohormones in apple diffusates is presented in ng/g of phosphate powder taken for the extraction of plant hormones after freeze-drying of apple diffusates (see Materials and Methods). For the explanation of GDH and FI, please refer to the Figure 2 in the main text.

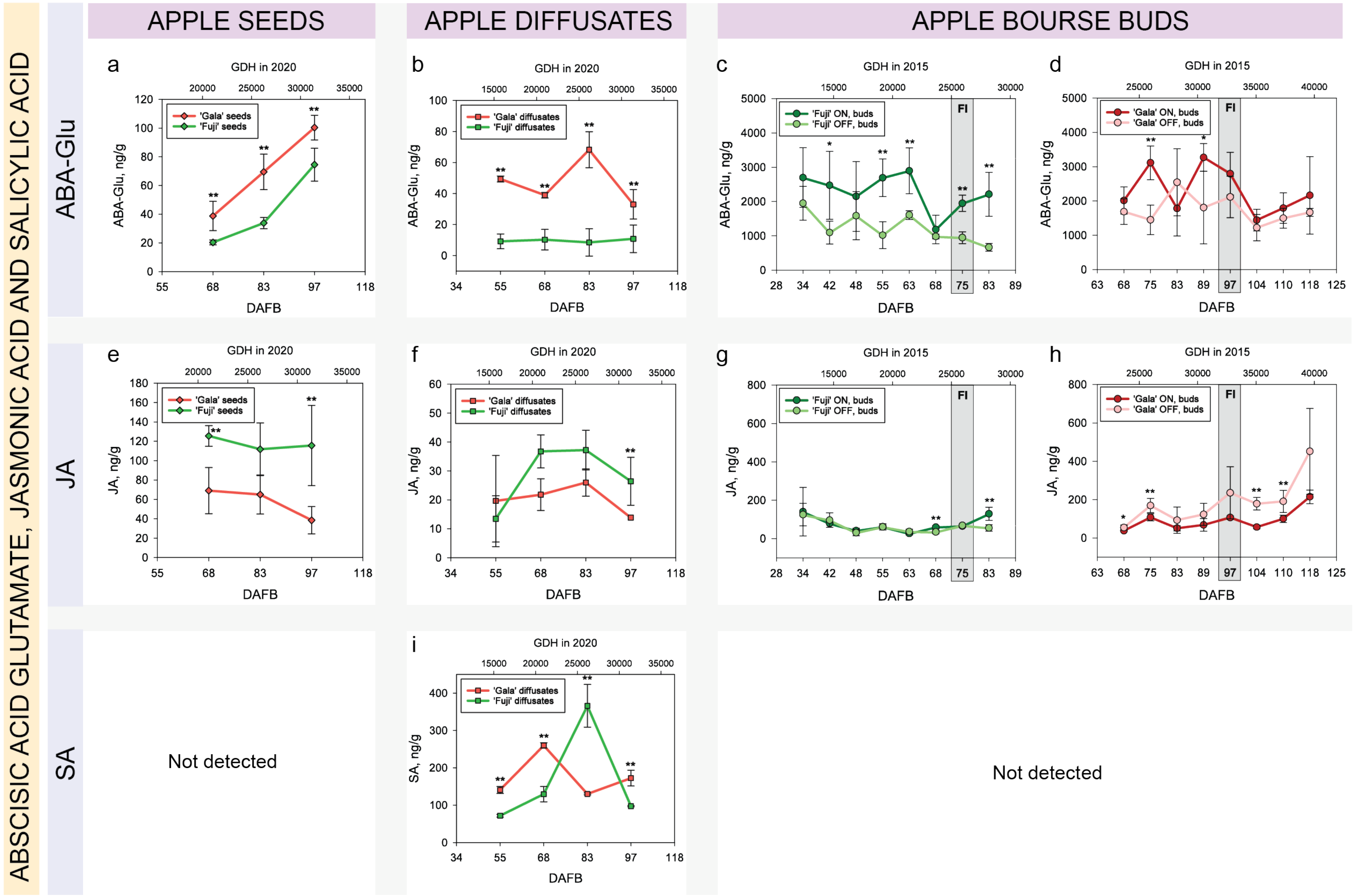

Supplementary figure 2. Concentration of ABA-Glu (a-d), JA (e-h) and SA (i) in seeds, fruit diffusates and bourse buds of 'Fuji' and 'Gala'.

Significant differences between cultivars and treatments are shown at p<0.05 (*) and p<0.01 (**). Concentrations of phytohormones in seeds and buds is specified in ng/g of FW. Concentrations of phytohormones in apple diffusates is presented in ng/g of phosphate powder taken for the extraction of plant hormones after freeze-drying of apple diffusates (see Materials and Methods). For the explanation of GDH and FI, please refer to the Figure 2 in the main text.

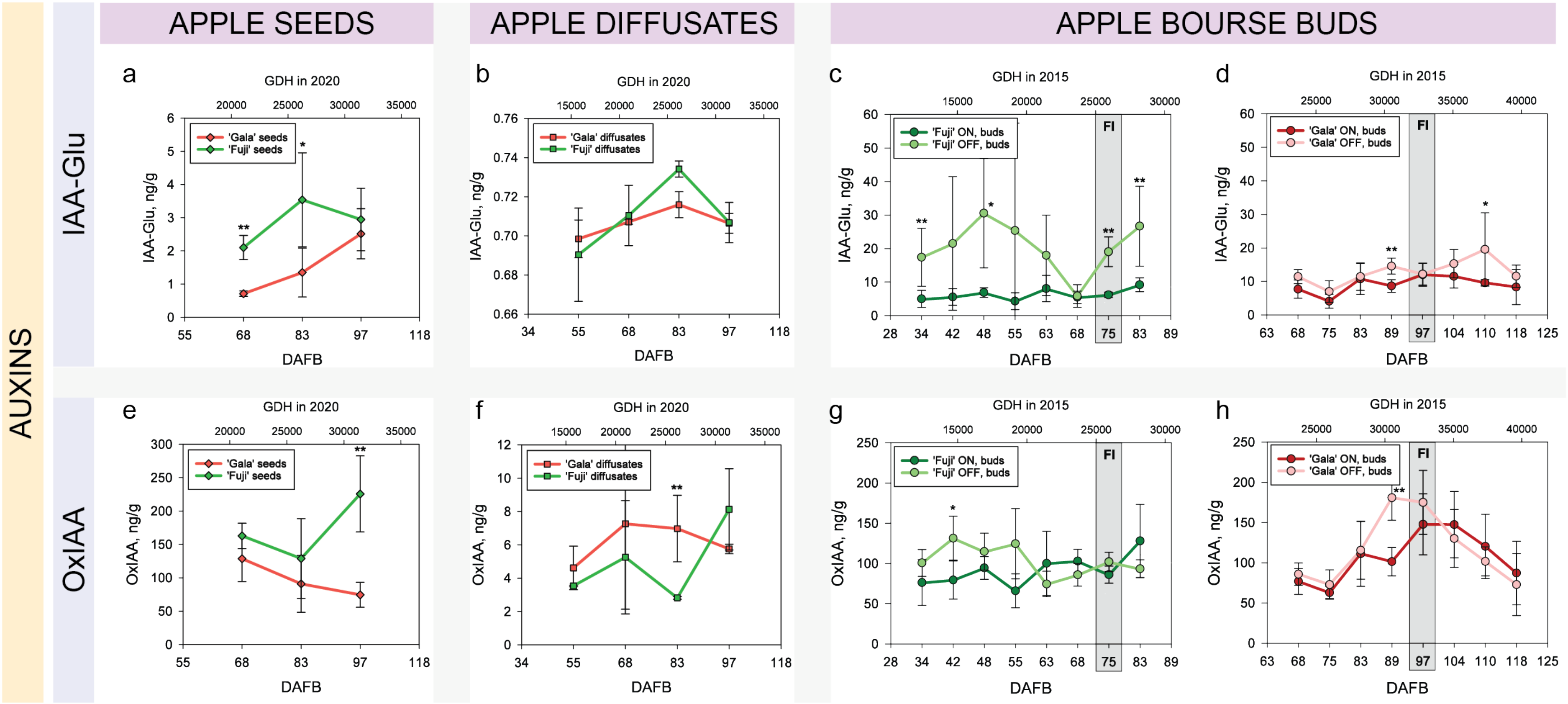

Supplementary figure 3. Concentration of IAA-Glu (a-d) and OxIAA (e-h) in seeds, fruit diffusates and bourse buds of 'Fuji' and 'Gala'.

Significant differences between cultivars and treatments are shown at p<0.05 (*) and p<0.01 (**). Concentrations of phytohormones in seeds and buds is specified in ng/g of FW. Concentrations of phytohormones in apple diffusates is presented in ng/g of phosphate powder taken for the extraction of plant hormones after freeze-drying of apple diffusates (see Materials and Methods). For the explanation of GDH and FI, please refer to the Figure 2 in the main text.
