# Supplementary material for: Profiling of phytohormones in apple fruit and buds regarding their role as potential regulators of flower bud formation
Source: Tree Physiol. 2022 Aug 11;42(11):2319–35. doi: 10.1093/treephys/tpac083 (PMC9912367; doi:10.1093/treephys/tpac083)
Supplement: Suppl_fig_4-8_tpac083 [file suppl_fig_4-8_tpac083.pdf]

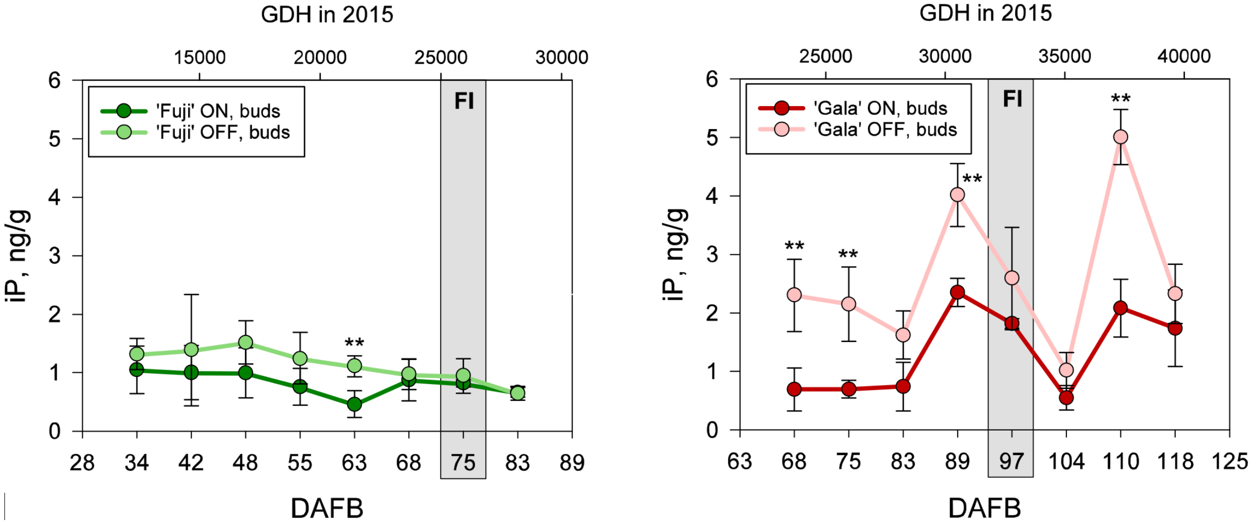

Suppl. Fig. 4. Concentration of iP in apple bourse buds of ‘Fuji’ (left) and ‘Gala’ (right).

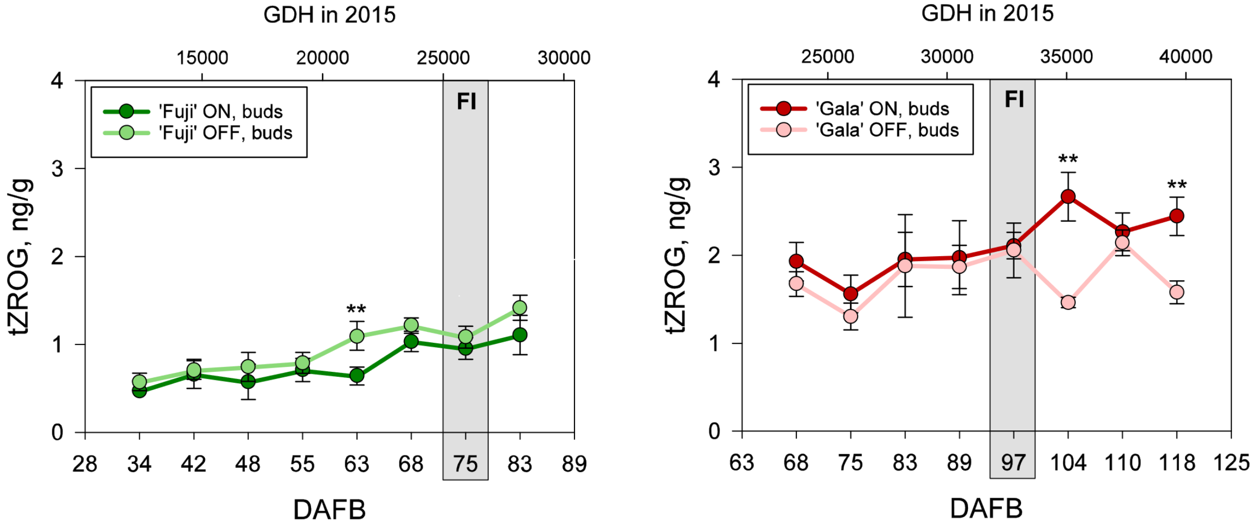

Suppl. Fig. 5. Concentration of tZROG in apple bourse buds of ‘Fuji’ (left) and ‘Gala’ (right).

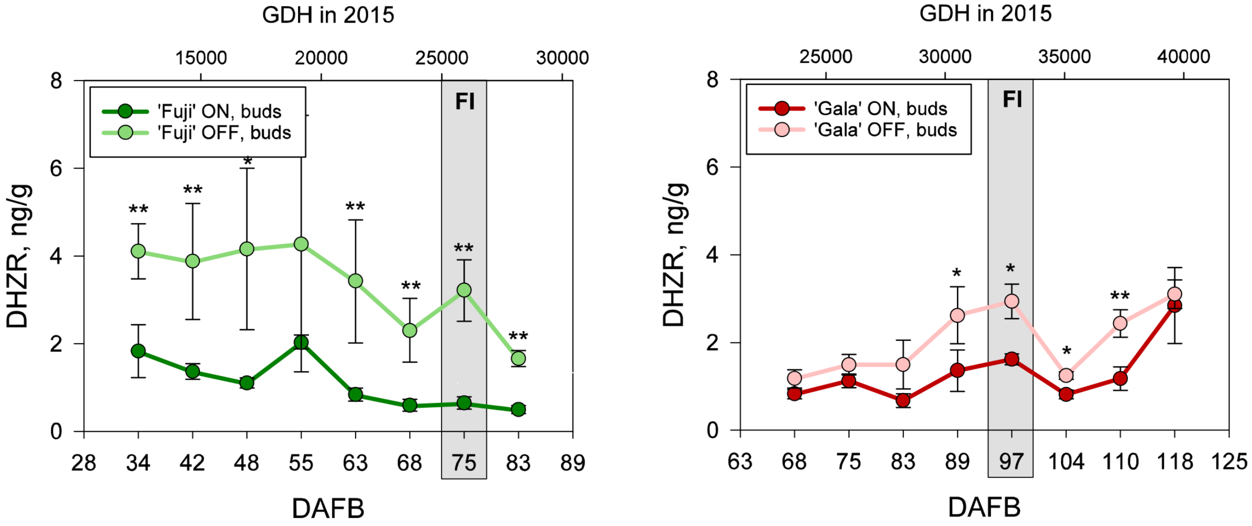

Suppl. Fig. 6. Concentration of DHZR in apple bourse buds of ‘Fuji’ (left) and ‘Gala’ (right).

* Significant differences between the buds from ON- and OFF-trees at $p<0.05$;

** Significant differences between the buds from ON- and OFF-trees at $p<0.01$;

The concentration is specified in ng/g of FW. For the explanation of GDH and FI, see Figure 2 of the publication.

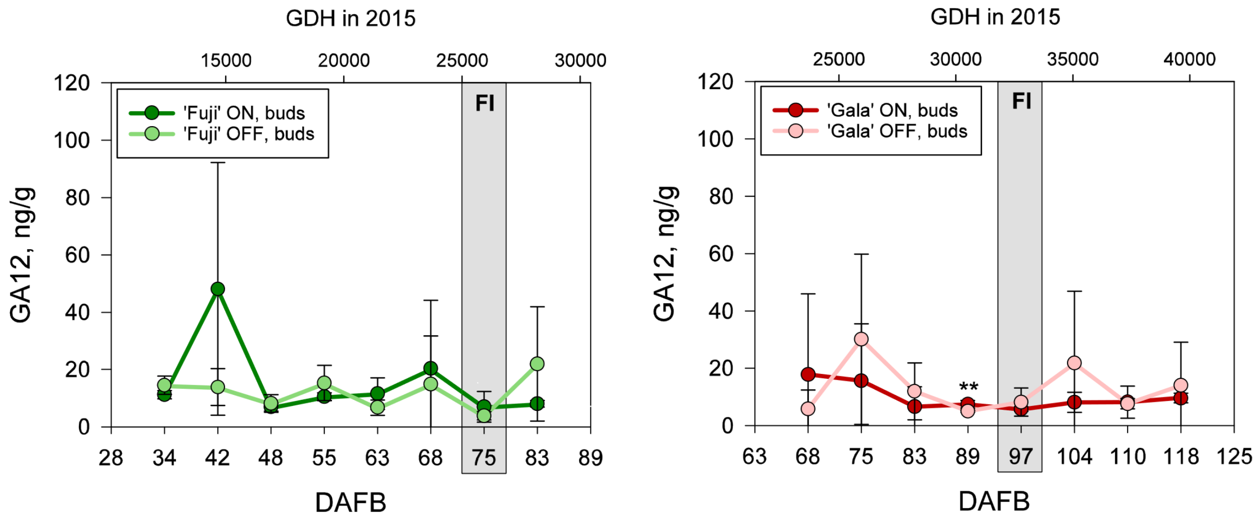

Suppl. Fig. 7. Concentration of GA12 in apple bourse buds of 'Fuji' (left) and 'Gala' (right).

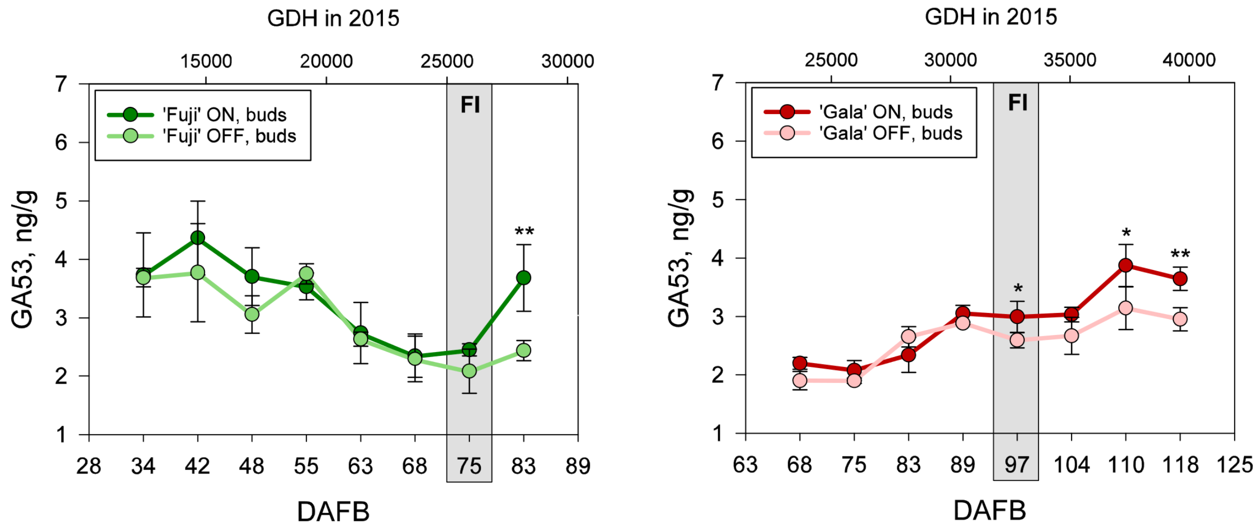

Suppl. Fig. 8. Concentration of GA53 in apple bourse buds of 'Fuji' (left) and 'Gala' (right).

* Significant differences between the buds from ON- and OFF-trees at $p<0.05$;

** Significant differences between the buds from ON- and OFF-trees at $p<0.01$;

The concentration is specified in ng/g of FW. For the explanation of GDH and FI, see Figure 2 of the publication.
