# Supplementary material for: Profiling of phytohormones in apple fruit and buds regarding their role as potential regulators of flower bud formation
Source: Tree Physiol. 2022 Aug 11;42(11):2319–35. doi: 10.1093/treephys/tpac083 (PMC9912367; doi:10.1093/treephys/tpac083)
Supplement: Suppl_fig_9_tpac083 [file suppl_fig_9_tpac083.pdf]

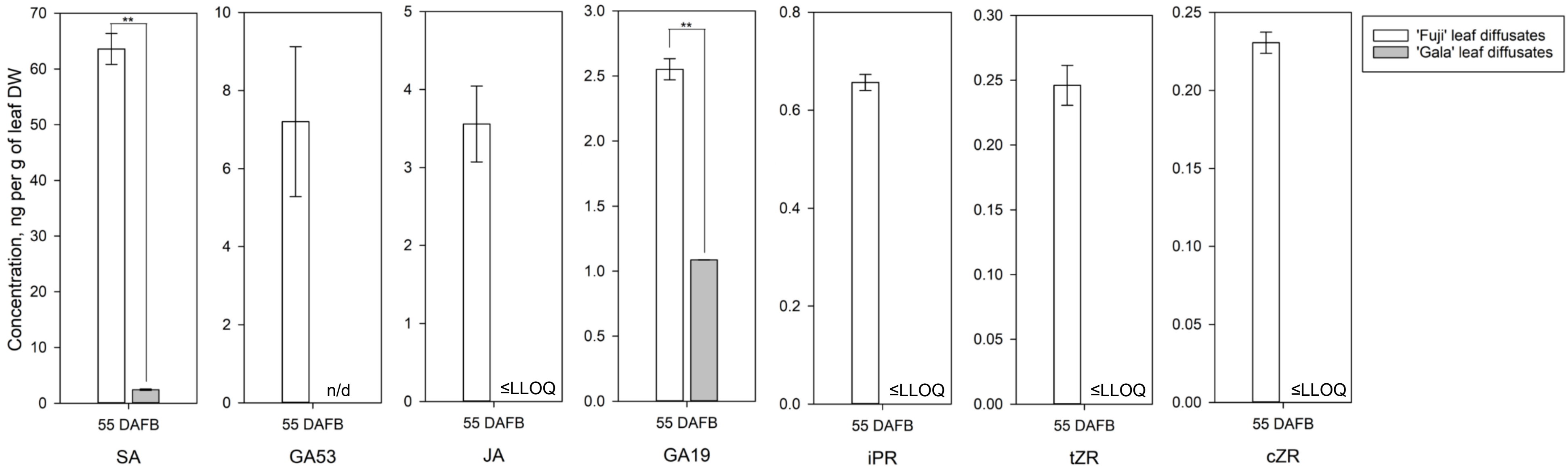

Suppl. Fig. 9. Concentration of phytohormones in leaf diffusates of ‘Fuji’ and ‘Gala’.

** Significant differences between the diffusates from ‘Gala’ and ‘Fuji’ at $p<0.01$;
n/d Not detected;
≤LLOQ Detected but below the lowest limit of quantification;

Concentrations of the compounds are specified in ng of each phytohormone exported to 2.5-ml phosphate buffer over 20 hours from 1 g DW of leaf material.
